# Nonsyndromic Bilateral Maxillary and Unilateral Mandibular Multiple Dentigerous Cysts in a Young Girl: Report of a Rare Case

**DOI:** 10.5005/jp-journals-10005-1082i

**Published:** 2010-09-15

**Authors:** L Krishna Prasad, P Srinivas Chakravarthi, M Sridhar, Y Ramakumar, Vivekanand Kattimani

**Affiliations:** 1 Professor and Head, Department of Oral and Maxillofacial Surgery, Sibar Institute of Dental Sciences, Guntur, Andhra Pradesh, India; 2 Professor, Department of Oral and Maxillofacial Surgery, Sibar Institute of Dental Sciences, Guntur, Andhra Pradesh, India; 3 Reader, Department of Oral and Maxillofacial Surgery, Sibar Institute of Dental Sciences, Guntur, Andhra Pradesh, India; 4 Postgraduate Student, Department of Oral and Maxillofacial Surgery, Sibar Institute of Dental Sciences, Guntur, Andhra Pradesh, India

**Keywords:** Dentigerous cyst, Bilateral maxilla, Unilateral mandible, Cuspids, Bicuspids, Incisor, Enucleation.

## Abstract

Dentigerous cyst is a benign odontogenic cyst associated with the crowns of permanent teeth. It is usually single in occurrence and located in the mandible of middle-aged persons. The teeth commonly affected are in order of frequency, the mandibular third molars, maxillary canines, maxillary third molars and, rarely central incisors. The present case report describes the surgical enucleation of the huge bilateral maxillary dentigerous cysts involving permanent maxillary canines associated with unilateral mandibular lateral incisor, cuspid and bicuspids in a young girl. To our knowledge, bilateral maxillary and unilateral mandibular multiple dentigerous cysts in a nonsyndromic patient have not been reported previously in the literature.

## INTRODUCTION

Dentigerous cyst is defined as ‘an epithelial-lined developmental cavity arising from the enamel organ due to an alteration in the reduced enamel epithelium and enclosing the crown of an unerupted tooth at the cementoenamel junction’.^1-3,5^ It develops from epithelial remnants of the reduced enamel organ as a result of fluid accumulation between its layers. This occurs due to obstruction of the venous outflow as a result of compression of tooth follicle by the erupting tooth.^3^ It is believed that epithelium, which is derived from the cell rests of Malassez lines the lumen of dentigerous cysts.^6,7^ Frequency of dentigerous cyst in the general population has been estimated at 1.44 cysts for every 100 unerupted teeth.^3^ Bilateral and multiple cysts have been reported in patients with syndromes or systemic diseases’ such as mucopolysaccharidosis and cleidocranial dysplasia.^1,3,8,10^ There have been only 19 cases of multiple nonsyndromic cysts reported in the literature from 1943 to 2005’^1^ but none of these presented with a differential incidence in both jaws as it is in our case. Here we presented very rare case of maxillary bilateral and mandibular unilateral dentigerous cysts first of its kind in the reported literature. This unusual case is one of nonsyndromic cysts involving maxillary cuspids bilaterally and unilateral mandibular lateral incisor’ cuspid’ and bicuspids in a 12-year-old girl.

## CASE REPORT

A 12-year-old girl presented with a right maxillary swelling of 4 months duration with a history of gradual enlargement of right jaw. On general examination’ the patient was apparently healthy. There was no significant past medical history. Intraoral examination revealed a bony swelling’ extending from the buccal vestibule of the maxillary right lateral incisor to the first deciduous molar. The swelling was well defined, firm in consistency, painless on palpation, and measured about 3 × 3 cm. The buccal cortical plate showed slight expansion and the overlying mucosa was normal. The radiological examination of the lesion in both jaws was associated with unerupted teeth. The computed tomography (CT) showed impacted maxillary canines bilaterally associated with radiolucent cystic lesions (Fig. 1A) and similar cystic lesion on the right side of mandible involving unerrupted lateral incisor, cuspid, and bicuspids (Fig. 1B). The 3D computed tomography (3D-CT) of facial bones showed perforation of the cortical plates bilaterally (Fig. 1C). Computed tomography (CT) showed obliteration of left maxillary sinus (Fig. 1D). Aspiration revealed the straw colored fluid. Histopathologically lesion was to be confirmed diagnosis of dentigerous cyst.

The cyst was enucleated under general anesthesia using Caldwell-Luc approach (Figs 2A and B) and crevicular incision in the mandible extending from lateral incisor to first molar on the right side. Cysts enucleated along with removal unerrupted lateral incisor, cuspid and bicuspids with deciduous molars in the mandible; bilateral cuspids in the maxilla. Intraoperatively on left side, cyst occupied whole of the left maxillary antrum containing a pale yellow fluid. The lining epithelium was found to surround the crowns of the unerupted cuspid and bicuspids with attachment at CEJ (Fig. 3) in maxilla and mandible respectively. Wound closure done after complete currattage, proper irrigation and hemostasis. On the 7th day, sutures were removed (Fig. 4). The patient was rescheduled for follow up. After 6 months, the patient was asymptomatic. Follow up OPG showed uneventful healing with an evidence of good bone fill (Fig. 5).

**Fig. 1A:**
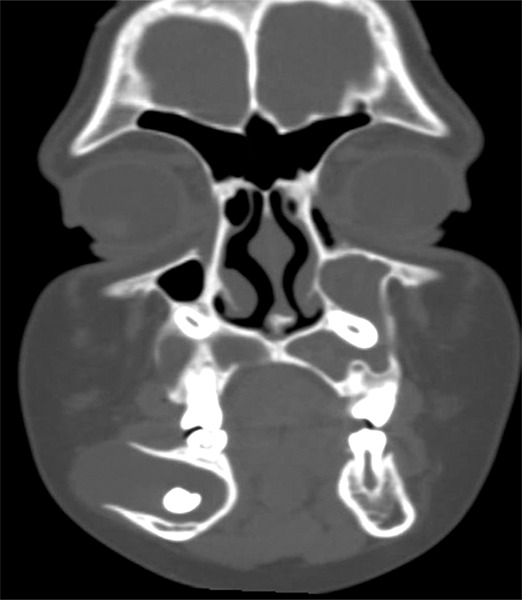
Coronal section of CT at first molar region showing impacted maxillary canines affecting sinus on both sides

**Fig. 1B:**
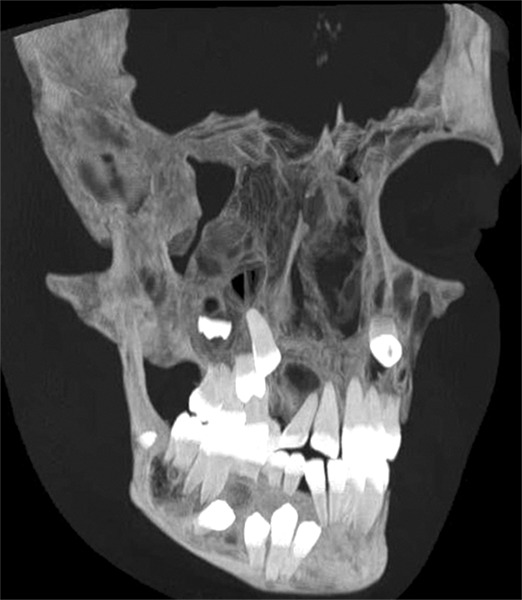
Radiographic view of CT facial bones showing impacted bilateral maxillary canines (13, 23) and mandibular teeth 42, 43, 44, 45

**Fig. 1C:**
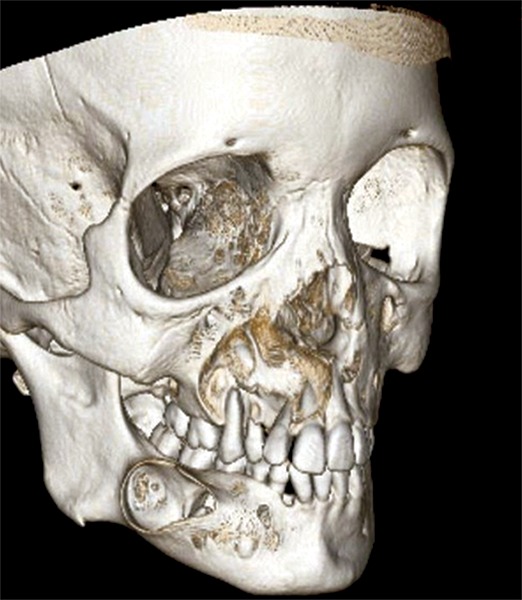
3DCT facial bones showing right lateral view of maxilla and mandible with cortical perforation involving periapical area of 12,14,15 and right maxillary sinus

**Fig. 1D:**
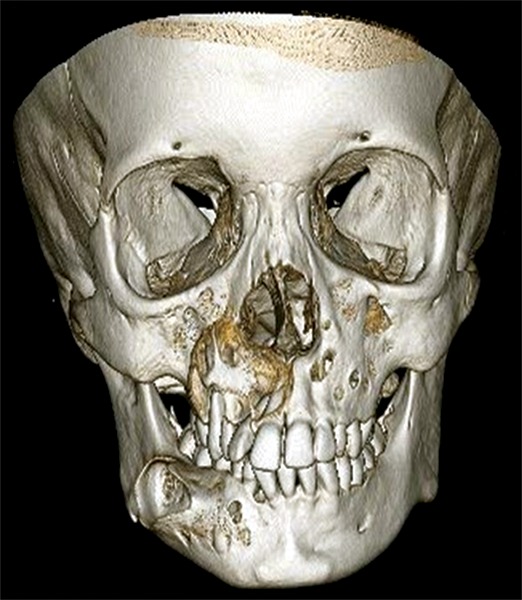
3DCT facial bones showing frontal profile with cortical perforation involving right and left maxillary sinus area and right body of mandible

## DISCUSSION

Dentigerous cysts are benign odontogenic cysts associated with the crowns of permanent teeth. Cysts involve impacted, unerupted permanent teeth, supernumerary teeth, odontomas, and, rarely, decidious teeth.^1^ They are usually seen in the second and third decades. When compared to females, the incidence is slightly higher in males.^1-3^ Mandible is likely to be the primarily affected site, as cysts are located in the mandible in 75% of the cases. The most frequently involved teeth are the mandibular third molars and maxillary canines.^3-5,7,9^ Dentigerous cysts are usually painless but may cause facial swelling and delayed tooth eruption. Usually pain or discomfort is not observed with the cysts unless they become secondarily infected. Thus, dentigerous cysts are frequently identified when radiographs are taken with the purpose to analyze a failure of tooth eruption, a missing tooth or malalignment. Simultaneous extensive maxillary and mandibular involvement and childhood presentation are rare^5,13^.

**Fig. 2A:**
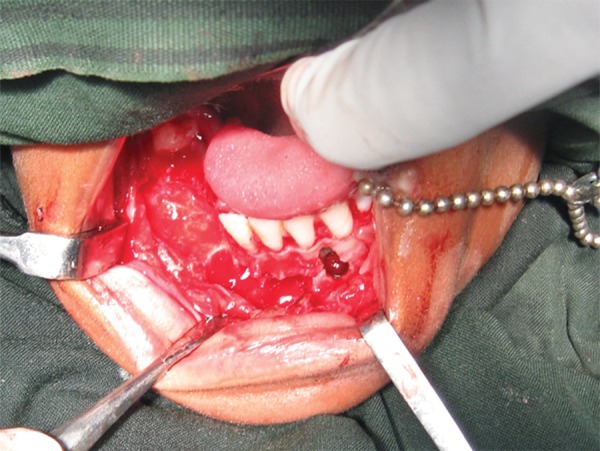
Intraoperative oral photograph showing bone defect in mandibular body after cystic enucleation with extraction of associated teeth (42, 43, 44, 45 retained 84, 85)

**Fig. 2B:**
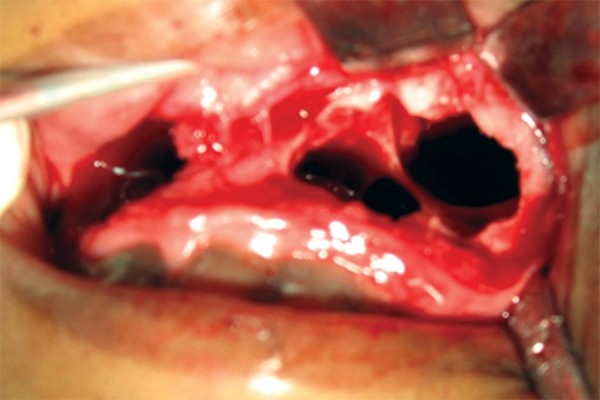
Intraoperative oral photograph showing bone defect in bilateral maxillary sinus area after cystic enucleation with extraction of involved teeth (13, 23)

**Fig. 3:**
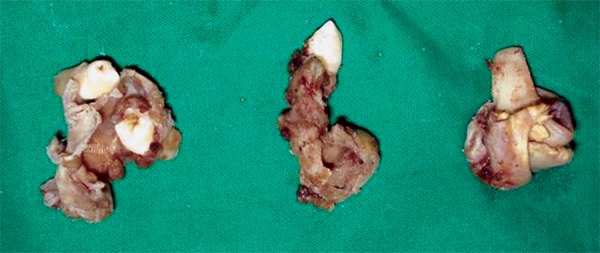
Cystic lining with associated teeth

Dentigerous cysts are usually solitary in occurence.^3,12^ Radiographic examination usually reveals a unilocular, radiolucent lesion that is associated with the crown of an unerupted tooth and characterized by well-defined sclerotic margins.^1-3,7-10^ Other odontogenic cysts like radicular cysts, odontogenic keratocysts, and odontogenic tumors, such as ameloblastoma, Pindborg tumor, odontoma, odontogenic fibroma, and cementomas may share the same radiologic features as dentigerous cysts.^1,4,14^ Microscopic evaluation is necessary most of the time to define the type of lesion.^4^

Bilateral and multiple cysts have been reported in patients with syndromes, such as basal cell nevus syndrome, mucopolysaccharidosis, and cleidocranial dysplasia.^3,6,7^ Bilateral mandibular dentigerous cysts have also been reported after prolonged concurrent use of cyclosporine and calcium channel blockers.^5^ Gingival hyperplasia and impaired dentition are the most common features shared by most of the syndromes.^3,5-7^

Till date 19 reported bilateral dentigerous cysts in nonsyndromic patients have been located in the mandible.^1^ One case of multiple dentigerous cysts involving both maxilla and mandible in a nonsyndromic patient with chickenpox has been reported.^9^ As with this case, nearly half of the patients were presented with painless facial swelling.^2,3,10^ Due to the presence of multiple dentigerous cysts, it was decided to further evaluate the patient for any possible syndromic association. For this purpose, a skeletal survey was performed to rule out the cysts in any other bone. This survey revealed no additional cysts. In this case, child is healthy with no abnormal physical or laboratory findings suggesting any syndromes.

**Fig. 4:**
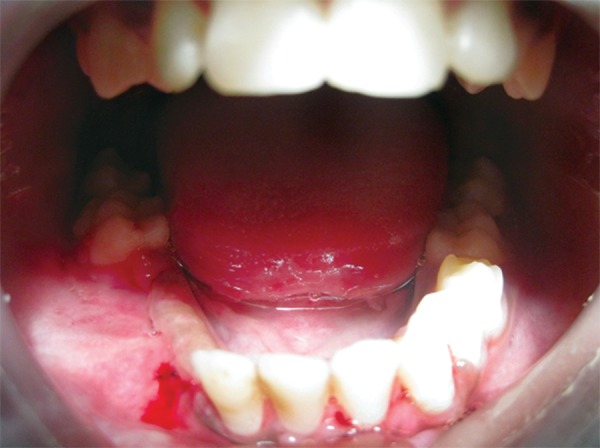
Intraoral postoperative photograph showing satisfactory wound healing

**Fig. 5:**
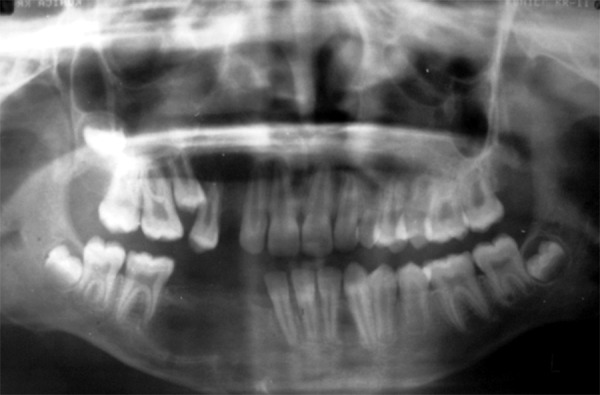
Postoperative OPG showing completely enucleated cystic lesions with extraction of associated teeth

In the maxilla, dentigerous cysts may be destructive and occupy the maxillary sinus; nasal cavities and even orbital encroachment may be observed.^4^ As with this case, maxillary cysts may displace and obliterate the maxillary antrum and nasal cavities. The cysts may cause fractures and become secondarily infected. Metaplastic and dysplastic changes may occur. An ameloblastoma, mucoepidermoid carcinoma, or squamous cell carcinoma may develop from the lining epithelium of a dentigerous cyst.^2,11,16^ Surgical excision and pathologic analysis of the lesion is essential for the definitive diagnosis.^1^

Treatment of dentigerous cyst depends on size, location, disfigurement and often requires variable bone removal to ensure total removal of the cyst, especially in the cases of large ones.^13^ This may even require Weber-Ferguson incision, as stated by Shah NJ.^15^ Scott-Brown has stated that marsupialization of the cystic lining is the treatment of choice for dentigerous cyst in children in order to give a chance to the unerupted tooth to erupt.^14^ But, in this case, as the tooth has been displaced into antral cavity far away from alveolar arch with a questionable viability and was unlikely to erupt on its own, so enucleation with removal of the displaced tooth was favoured.^12^ Moreover, as the enlarging cyst had occupied and already lined the antral cavity, its marsupialization would lead to an oroantral fistula with consequent antral sinusitis, so enucleation of the cyst with primary closure was chosen. The patient was followed up for 6 months. Overall symmetry of both sides of the face was observed with patency of both the nasal airways.

In conclusion, the occurrence of multiple cysts is a rare condition in nonsyndromic patients and there is no definitive predictive pattern of occurance. Present case reflects one such situation where the treatment had to be differed from the usual protocol mentioned in the literature making it a unique case of its kind.
